# Mesenchymal Stem Cells and Tuberculosis: Clinical Challenges and Opportunities

**DOI:** 10.3389/fimmu.2021.695278

**Published:** 2021-07-22

**Authors:** Xueying Zhang, Qi Xie, Ziyu Ye, Yanyun Li, Zhengping Che, Mingyuan Huang, Jincheng Zeng

**Affiliations:** ^1^ Dongguan Key Laboratory of Medical Bioactive Molecular Developmental and Translational Research, Guangdong Provincial Key Laboratory of Medical Molecular Diagnostics, Guangdong Medical University, Dongguan, China; ^2^ Institute of Laboratory Medicine, School of Medical Technology, Guangdong Medical University, Dongguan, China; ^3^ Dongguan Key Laboratory of Environmental Medicine, School of Public Health, Guangdong Medical University, Dongguan, China; ^4^ Key Laboratory of Medical Bioactive Molecular Research for Department of Education of Guangdong Province, School of Basic Medicine, Guangdong Medical University, Dongguan, China; ^5^ Collaborative Innovation Center for Antitumor Active Substance Research and Development, School of Basic Medicine, Guangdong Medical University, Zhanjiang, China

**Keywords:** mesenchymal stem cells, tuberculosis, granuloma, immunomodulation, application

## Abstract

Tuberculosis (TB) is one of the communicable diseases caused by *Mycobacterium tuberculosis* (Mtb) infection, affecting nearly one-third of the world’s population. However, because the pathogenesis of TB is still not fully understood and the development of anti-TB drug is slow, TB remains a global public health problem. In recent years, with the gradual discovery and confirmation of the immunomodulatory properties of mesenchymal stem cells (MSCs), more and more studies, including our team’s research, have shown that MSCs seem to be closely related to the growth status of Mtb and the occurrence and development of TB, which is expected to bring new hope for the clinical treatment of TB. This article reviews the relationship between MSCs and the occurrence and development of TB and the potential application of MSCs in the treatment of TB.

## Introduction

Tuberculosis (TB) is an infectious disease caused by *Mycobacterium tuberculosis* (Mtb) infection, which is mainly transmitted through the respiratory tract. According to the *World Health Organization’s Global TB Report 2020*, it is estimated that 10 million people worldwide will be affected by TB in 2019, with 7.1 million new cases. In addition, nearly half a million people suffer from rifampin-resistant tuberculosis (RR-TB), of which 78% suffer from multidrug resistant tuberculosis (MDR-TB). Despite continuous progress in TB diagnosis and effective drug treatment, about 1.21 million people still died of TB in 2019, and the gap between the reported number of registered TB patients and the estimated number remains large (1). During Mtb infection, Mtb may invade various organs throughout human body, it is generally believed that Mtb can be transmitted through atomized particles, and infectious droplets particle size range is generally 0.65 to 7 µm. Droplets below 0.65μm are mainly deposited in the distal airway through the nasopharynx or tracheobronchial area. The droplets with larger particle size were deposited in the upper respiratory tract or oropharynx, causing TB in oropharynx or cervical lymph node (2). Once in the lower respiratory tract, Mtb is mainly devoured by macrophages, while the inflammatory response causes a large number of immune cells to attract to the infected site, hyaluronic acid-mediated macrophage aggregation, then macrophage and its evolutionary cellular limitation immersion and proliferation of the state of clear nodule lesions, results the formation of tuberculosis granuloma (TG), which is the pathological mark of TB (3, 4). At the same time macrophages and fibroblasts, endoskines, and neutrophils produce a large number of proteases (metal proteases, lysosome proteases, as well as fibrosygen/fibrosase systems and their activators urine kinases) to promote the formation of TG by mediating antigen processing, forming extracellular matrix (ECM) and cell fragments, and processing cytokines and hormones (5, 6). The formation of TG is a key event to prevent the spread of infection and inflammation, during this period, Mtb can escape the host immune response and remain dormant for decades (7). Once the host’s immune response is weakened or suppressed, dormant Mtb may be reactivated and escape from TG, causing lesions in other parts of the tissue (8). Mtb can coexist with the host, but the mechanism of its resistance to the host’s strong immune response remains uncertain.

Mesenchymal stem cells (MSCs) are pluripotent stem cells, which adhere to plastic under standard culture conditions *in vitro*, showing long spindle and fibroblastic appearance, expressing mesenchymal surface markers CD29, CD73, CD90 and CD105, but not expressing hematopoietic stem cell surface markers CD11b, CD14, CD34, CD45 and HLA-DR (9). MSCs have been confirmed to exist in bone marrow, fat, skin, thymus, placenta, gingival, umbilical cord tissue, etc., and can be induced to differentiate into osteoblasts, adipocytes, chondroblasts and neuron cell under certain conditions (10, 11), etc. In addition, a number of studies have shown that MSCs can migrate to the site of injury in the body and effectively play the role of anti-inflammatory immune regulation and tissue regeneration by differentiating into damaged tissues and paracrine cytokines (12, 13), which have been widely used in the field of immune and inflammatory diseases and regenerative medicine (14, 15). Almost everything that is known about the lifestyle of Mtb in host cells of TG mainly through the study of macrophages. But recent studies have found that the occurrence and development of TB seems to be closely related to MSCs.

## MSCs and TG

### MSCs Regulates Mtb Growth

TG is an aggregate of cells composed of several types of infected and uninfected phagocytes (macrophages, monocytes, dendritic cells, and neutrophils) and T lymphocytes, and is the hallmark histopathological structure of TB (16). It represents the niche of long-term persistence of Mtb as well as host sequestration of bacteria in order to limit dissemination. To better understand pathogenesis of TB requires tackling its epicenter, the granuloma. More and more studies have found that MSCs are involved in formation and development of TG. Raghuvanshi et al. used CD29 as the identification marker for MSCs and found that MSCs were distributed over human TG and there were active acid-fast bacteria in the MSCs cluster. In addition, MSCs seem to play a complex role in granuloma. On the one hand, MSCs can produce the same specification of nitric oxide (NO) to inhibit T cell response with limiting the human immune response to kill Mtb, on the other hand, NO can inhibit the growth of Mtb and limit the bacteria within the granuloma (17), the persistent infection of Mtb *in vivo* and the formation of TG may be related to this mechanism. Moreover, Das et al. found that MSCs can be used as “niches” for Mtb dormancy in TG, because the live Mtb can be isolated from CD271^+^/CD45^-^ bone marrow mesenchymal stem cells (BM-MSCs) of TB patients that have successfully completed anti-TB treatment for several months (18). Hypoxia is known to cause Mtb dormancy (19), and Garhyan et al. found that Mtb containing CD271^+^MSCs are elevated in human subjects who have previously been treated for pulmonary TB, and proves the potential location of Mtb containing CD271^+^MSCs in the hypoxic niche (20). Interestingly, CD271^+^MSCs function and activity were significantly increased in adults or older adults compared to children (21), suggesting that CD271^+^MSCs may be closely related to the occurrence and development of TB. A recent study found that Mtb expressed dormancy related genes in CD45^-^Sca1^+^MSCs that sorted from bone marrow of infected mice (22). These evidences further confirm that MSCs are the natural host of latent Mtb infection. Additionally, recent studies have found that MSCs do exist in human pulmonary and extrapulmonary TG (23).

Besides, studies have shown that MSCs do regulate the growth of Mtb. Schwartz et al. found that MSCs could promote the growth of Mtb in splenic granuloma, while MSCs treated with Poly (A: U) could inhibit the growth of Mtb in splenic granuloma (24). In addition, MSCs display a novel phagocytom-like function and restrict Mtb growth *via* scavenger receptors (SR)-mediated internalize or phagocytosis and autophagy during TB infection (25). After MtB infection, MSCs also paracrine prostaglandin E2 (PGE2) and mediate Mtb resistance by affecting the bacteriophilic properties of MSCs (23). Notably, the metabolic activity of Mtb in MSCs will be at a low state and will gradually acquire resistance to first-line anti-TB drugs (26).

It has been reported that altruistic stem cells (ASCs) exhibit niche regulation or altruistic stemness in Mtb infected microenvironment of hypoxia/oxidative stress, and thus act as a niche defense mechanism (27). Therefore, dormant Mtb in cells may promote the reprogramming of MSCs in the lungs of acute respiratory infection (ARI) to the ASCs phenotype. The reprogrammed ASCs can then stimulate the replication of dormant Mtb and subsequently release them to neighboring MSCs and/or macrophages (28). This process will lead to primary TB in the lungs. Whereas in a coronavirus infected mouse model, dormant Mtb reprogrammed host CD271^+^MSCs into ASCs phenotype, which enabled the dormant bacteria to reactivate (18). Also, MSCs carrying live Mtb can be reengineered into ASCs by producing extracellular vehicles (EVs) that secrete Mtb antigens including ESAT-6 (29). EVs are disseminated through aerosols in the community and come into contact with mucosal immune cells of healthy subjects, leading to natural inoculation (29). These reports suggest that MSCs can regulate Mtb growth, mediate bacterial resistance, and involve in the formation of granuloma. Therefore, clarifying the status of bacteria and host cells in TG will greatly improve our understanding of the pathophysiology of TB and promote the development of new treatment methods.

### MSCs Promotes Fibrin Deposition

Fibrin deposition is crucial in TG. TGF-β is the primary activator of fibroblasts, and plays a key role in promoting wound healing. It stimulates muscle fiber cells to enhance repaired wounds and produces ECM to support the formation of granulation tissue (30). Additionally, the highly expression of tissue factor (TF) on the surface of MSCs is an activator of the exogenetic clotting pathway, in which the expression of TF and the ability to activate local coagulation triggers the activation of fibrin and leads to fibrin deposition required for TG formation (31–33). It provides a new idea for the study of the mechanism of MSCs-mediated TG formation.

### Possible Forms of MSCs in TG

TG is an aggregate of a variety of immune cells, and its unique environment may affect the biological functions of MSCs. However, there are few studies on the relationship between MSCs and TG. It is still unclear which form of MSCs exist in TG and what role they play. Mature adipocytes from adipose tissue can inhibit the accumulation of lymphocytes and neutrophils, but the destruction of normal tissue matrix homeostasis, such as chronic inflammatory diseases, may lead to “abnormal” lipogenesis. Studies have shown that MSCs-differentiated adipocytes lost their ability to inhibit neutrophils from adhering to TNF-α stimulated endothelial cells in co-culture system with human umbilical vein endothelial cells but had the ability to inhibit lymphocyte adhesion (34). Chen et al. found for the first time that lysophosphatidic acid (LPA) had an abnormally high abundance in the plasma of untreated patients with TB, but with the progression of treatment, the abundance of LPA significantly decreased (35). LPA activates intracellular transcription factor peroxisome proliferator-activated receptor-γ (PPAR-γ) (36), during Mtb infection PPAR-γ induce accumulation of lipid droplets (LDs) in foamy macrophages (37, 38). Interestingly, PPAR-γ is recognized as a major inducer of adipogenesis, regulating the differentiation of MSCs into adipocytes (39). In addition, adipose tissue has been proposed as an extrapulmonary niche in which Mtb can exist for a long time (40). Therefore, we speculate that in TG, MSCs may partially differentiate into adipocytes, which on the one hand provide the energy needed for the survival of Mtb, and on the other hand reduce the damage of Mtb caused by immune reaction by inhibiting lymphocyte adhesion.

Multinucleated giant cell (MGC) is the characteristic cells of TG, but the formation mechanism and role of MGC are still controversial. Most views believe that MGC originates from the fusion between monocytes and macrophages (41). Another view suggests that MGC may be formed from epithelioid macrophages during mitosis or without cytoplasmic division during amitosis (42, 43). Notably, recent research has found that some MSCs tend to fuse and form multinucleate cells at 5 days after Mtb infection (25). But whether the formation of MGC is related to MSCs remains to be further confirmed.

Studies have found that local delivery is known to cause MSCs to aggregate into “spheroids,” This aggregation phenomenon has been observed in rodents after intraperitoneal and subcutaneous (13, 44), and intraventricular (45) injections. Although adherent MSCs can effectively inhibit T cells, the aggregation of MSCs into globules will result in the loss of their ability to inhibit T cells and enhance their anti-inflammatory ability (46, 47). TG is a collection of various cells. It is not clear whether MSCs converge in this space-restricted location, and whether the accumulation of MSCs affects their own functions. We believe that MSCs may have multiple “identities” in granulomas, but further verification is needed.

### Physical Location of MSCs in TG

**Figure 1:**
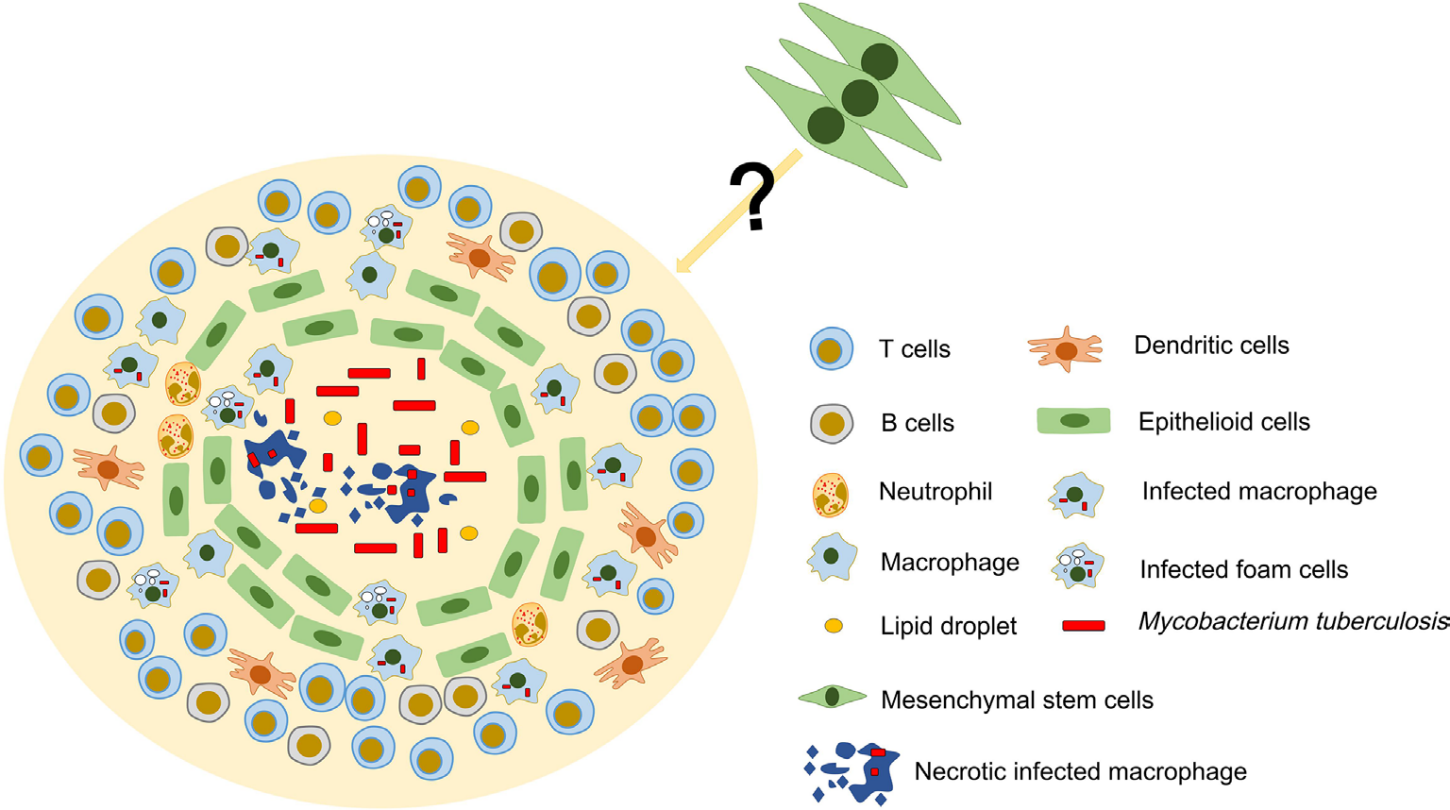
Possible structure and cellular constituents of TG. TG is an organized aggregate of immune cells. In classic TG, there is a necrotic area in the center, surrounded by a large number of immune cells such as T cells, B cells and macrophages. MSCs have been found to exist in TG and may be involved in the formation and development of granuloma. However, the location of MSCs in TG and their relationship with immune cells are still unclear.

The classical TG exhibits the following structures. Central acellular necrotic core, surrounded by a variety of macrophages that is itself circumscribed by a lymphocytic cuff of T cells and B cells, and may have a peripheral fibrotic edge (16). As a “new member” in TB, it is not clear whether MSCs have a specific physical location in TG. It was found that MSCs surrounds the granulomatous structure and inhibits T cell activation and restricts the growth of Mtb by producing NO. This is due to the fact that MSCs is very close to living Mtb and IFN-γ-producing T cells, thus creating an environment conducive to the production of NO by these cells (17). Therefore, we guess that MSCs are not disorganized, but have a specific physical location distribution between them and the host immune cells in the TG. In addition, the lymphocyte-rich area of the granuloma is usually located in the outer layer, and this distribution may limit the interaction of T cells with infected macrophages that are mainly located in the inner area of the granuloma (48). However, the immunomodulatory effects of MSCs and their distribution in granulomas and whether they are affected by the distribution of lymphocytes are not yet fully understood (**Figure 1**). In general, determining the distribution of MSCs may be a key step in exploring the formation mechanism of TG.

## Potential Application of MSCs in TB

### MSCs as Potential Therapeutic Targets

It has been found that cytokines such as IFN-γ and TNF-α activate macrophages and promote the secretion of oxidants such as nitric oxide and superoxide, thereby killing Mtb (25). However, the bacterial colony forming unit (CFU) increased in a dose-dependent manner after IFN-γ and TNF-α were applied to Mtb infected MSCs (23). Several vaccine candidates rely on CD4^+^ and CD8^+^T cells that secrete IFN-γ well (49–51). These vaccines may be effective on macrophages instead of Mtb in MSCs, so the efficacy may be affected. Lipid droplets(LDs) are independent organelles composed of lipid ester nuclei and a surface phospholipid monolayer, which are involved in various cellular functions (52). Recent studies have shown that Mtb in MSCs induces lipid synthesis and hides within LDs, thus impeding antimicrobial host defense mechanisms, while inhibition of lipid synthesis prevents dormancy and sensitized the organisms to isoniazid (22). Notably, Knight et al. found that IFN-γ/HIF-1α signaling drives LDs formation in macrophages, and that LDs formation is inversely correlated with bacterial lipid acquisition and growth (53). Although the mechanism of LDs formation in MSCs remains unclear, we believe that the mechanism of LDs formation in MSCs may be independent of IFN-γ. In a word, the mechanism of lipid synthesis induced by Mtb in MSCs should be clarified. By effectively inhibiting the lipid synthesis of MSCs, cutting off the energy supply of Mtb, and activating the host defense mechanism, it is of great significance for the treatment of TB. Current TB treatment relies on a combination of antibiotics. However, antibiotics focus on the elimination of replicating rather than dormant Mtb (22). There is an urgent need to find new ways to eliminate persistent bacteria. Interestingly, recent research has found that MSCs are natural reservoir for latent Mtb infection, whereas macrophages support the replicating form of Mtb (22). Therefore, understanding the mechanism by which Mtb adapts to MSCs and the targets of MSCs allowing the long-term existence of Mtb is the key to achieve sterilizing TB cure. In the process of TB infection, the intracellular events of macrophages and MSCs are different (**Table 1**). Understanding the differences in macrophages and MSCs after Mtb infection may help to achieve this goal.

**Table 1:** The differences between macrophages and MSCs after Mtb infection.

Item	Macrophages	MSCs
LDs	IFN-γ/HIF-1α promotes the formation of intracellular LDs (53); lower levels of LDs accumulation (26)	Mechanism is unknown; high levels of LDs accumulation (26)
IFN-γ stimulation	Activating macrophages to kill Mtb; The expression of ROS increased; No increase in PGE2 release (54)	The CFU of intracellular Mtb increased in a dose-dependent manner; ROS production is not affected; The expression of PGE2 increased; increasing autophagy flux (23)
Phagocytosis of Mtb	Mannose receptor (MR) is a major receptor (55)	MR was not involved (25)
Autophagy	Decreasing Mtb viability (56) Autophagy is induced by activated macrophages (56, 57)	Intracellular Mtb growth restriction; MSCs have inherent autophagy (25)
Intracellular Mtb	Active replication (22); no significant change in morphology (26); most of Mtb are located to early endosomes immediately after infection (22)	No proliferation and dormancy state (22, 25); increasing bacterial cell density and reduction in cell size (26); Most of Mtb are located in cytosol (22)
Cellular states	Cells die at very low levels of infection (26)	Cells enter into a quiescent state (22)
Oxidizing reaction	Releasing lower levels of NO (58)	Releasing higher levels of NO (25)
Phagocytic ability of Mtb	Stronger (22)	Weaker (22)
Mtb efflux pumps	Rv0194, Rv1218c, Rv1272c, Rv1273c, Rv1463, Rv1687c, Rv2686c, Rv2687c, Rv2688c, Rv1348, Rv1349, Rv3239c, Rv3728, Rv1183, Rv1146, Rv0969, Rv3578 (59)	ABC transporters ABCC1 and ABCG2 (23)

### Therapeutic Potential of MSCs in TB

#### MSCs as Modulators of Immune Cells

It is known that the emergence of MDR-TB and the pandemic of novel coronavirus in recent years have posed a major threat to global TB prevention and control (60, 61). Immune dysfunction in TB patients and the emergence of Mtb resistant strains lead to unsatisfactory therapeutic effects, so it is critical to find a new therapeutic regimen. It is considered that the incidence of TB is largely dependent not on primary infection or re-infection, then on the reactivation of persistent latent Mtb due to immunodeficiency (62). Therefore, it is essential to improve the body immunity for TB treatment. In recent years, host-directed therapy (HDT) has emerged as an emerging approach, especially in chronic pathogen infections such as TB. HDT aims to improve the control of infection and the alleviation of inflammation by modulating the host immune response, thus reducing tissue damage (63). MSCs have attracted wide attention due to their powerful immunomodulatory functions and their ability to replace or repair damaged tissue and may be ideal candidates for chronic diseases.

**Figure 2:**
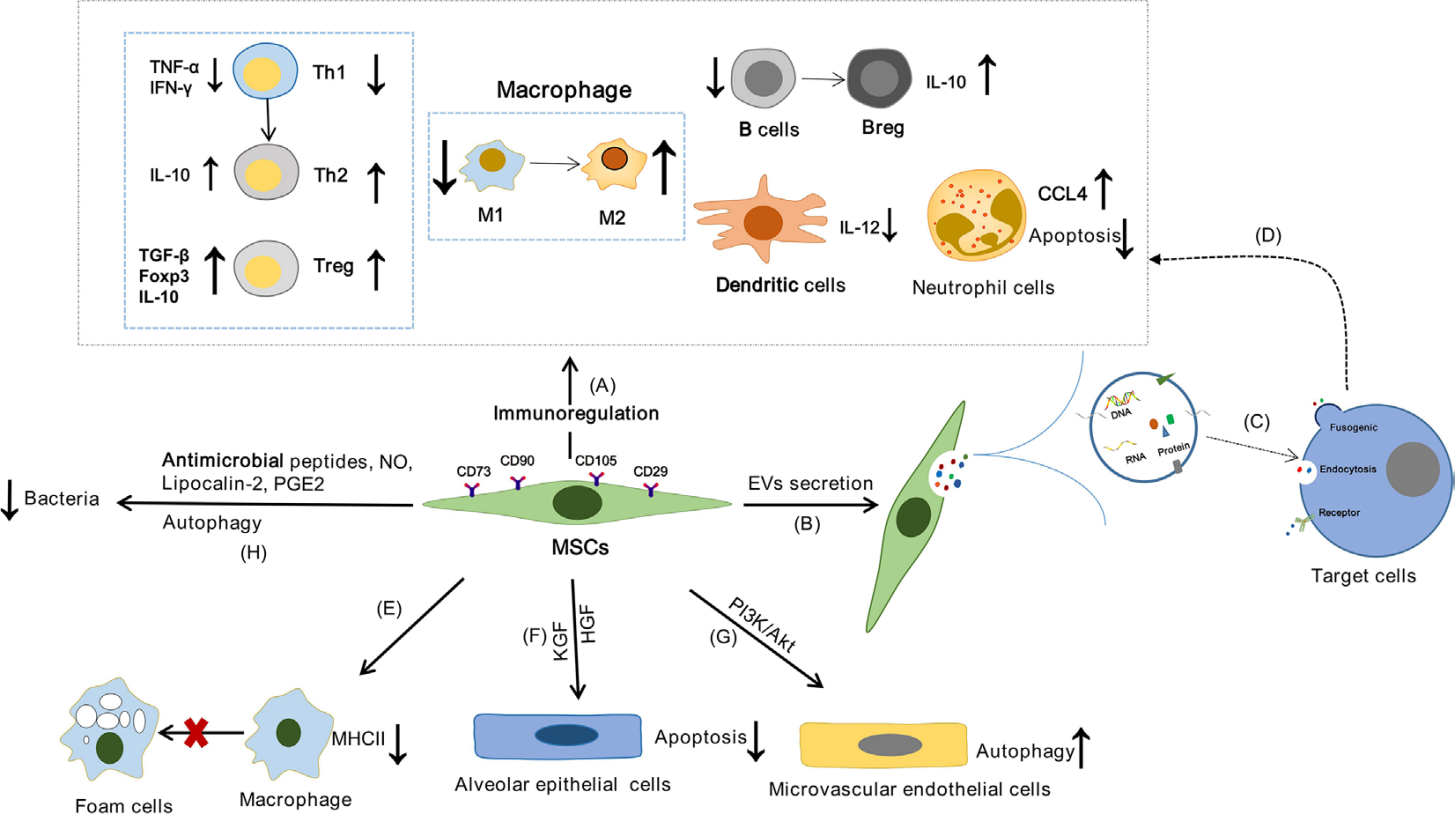
The possible mechanism of MSCs in the treatment of TB. MSCs may achieve the purpose of treating TB by regulating the inflammatory response of TB, alleviating the lung injury of TB, affecting the survival of host cells and clearing bacteria. **(A)** The functions of innate immune cells and adaptive immune cells can be effectively regulated by MSCs through “direct interaction between cells”, secreting enzymes and cytokines. **(B)** EVs secreted by MSCs are lipid vesicles that mediate intercellular communication. **(C)** EVs can be used as antigen to bind to the target cells, or as a carrier to deliver proteins, DNA, mRNA and non-coding RNA to the target cells. **(D)** The function of immune cells can be regulated by MSCs through the release of EVs. **(E)** Macrophages can differentiate into foam cells, which are characterized by lipid accumulation. And MSCs can affect the lipid homeostasis of macrophages, inhibit the formation of foam cells, and reduce the expression of MHCII in macrophages, thereby reducing the uptake of oxidized low density lipoprotein. **(F)** KGF and HGF secreted by MSCs protect alveolar epithelial cell from apoptosis under hypoxic conditions. **(G)** MSCs can enhance pulmonary microvascular endothelial cell autophagy through PI3K/Akt signal transduction. **(H)** MSCs exhibited antimicrobial activity by secreting antimicrobial peptides and NO, enhancing the activity of lipoprotein-2, and enhanced autophagy.

MSCs can affect the immune system of human body through “direct interaction between cells”, enzyme production and soluble cytokines. It has been reported that MSCs can promote the functions of Tregs and Th2, inhibit the release of IFN-γ by Th1 and regulate the balance of Th1/Th2 though PGE2 secretion (64). In addition, MSCs can regulate T cell response and promote the polarization of M1 to M2 macrophages by expressing indoleamine 2,3-dioxygenase (IDO) and activating the CD39/CD73/adenosine signaling pathway (65, 66). FasL expressed by MSCs may bind to Fas expressed by activated T cells to up-regulate Treg and promote T cell apoptosis through the Fas/FasL signaling pathway (67). In addition, PGE2 released by MSCs can inhibit the maturation and function of dendritic cells (DCs), resulting in a decrease in IL-12 expression (68). MSCs can inhibit the activation of B cells and promotes the transformation of B cells into IL-10-producing Breg, and IDO partially participates in the MSCs-mediated effects on Breg cells (69, 70) (**Figure 2A**). In addition, the effect of MSCs has been attributed to cell-to-cell communication released by EVs (**Figure 2B**). EVs are lipid vesicles secreted by cells that mediate intercellular communication. According to their size and intracellular origin, EVs can be divided into exosomes, microvesicles and apoptotic bodies (71). EVs can be used as antigen to bind to recipient cells and can also be used as a carrier to deliver functional substances such as protein, DNA, mRNA and non-coding RNA to recipient cells (**Figure 2C**). Studies have shown that MSCs-derived extracellular vesicles (MSCs-EVs) also have strong immunomodulatory ability (**Figure 2D**). MSCs-EVs can down-regulate Th1 response *in vitro*, reduce the number of Th17 cells and IL-17 secretion, and increase the proportion of Treg cells (72). They also affect B cell lineage function by inhibiting immunoglobulin secretion (73). In addition, MSCs-EVs can be effectively internalized by mononuclear macrophages, promote the polarization of M0 macrophages to M2, inhibit the expression of CCR7 in DC cells and weaken the function of DC cells by carrying miR-21-5p (74, 75). In a word, MSCs-EVs can produce a wide range of immunomodulatory effects on different cells of the immune system.

#### Therapeutic Theories of Immunomodulatory

T cells are known to play a key role in the host’s immune response against Mtb infection. It has been found that T cells are necessary for long-term control of Mtb infection and may play an important role in the prevention of TB (48, 76). However, a strong T-cell response is not necessarily beneficial, and TB patients must strike a fine balance between immune system activation and tolerance. In healthy people, Th1 and Th2 cytokines are in a dynamic equilibrium state. During Mtb infection, Th1 cytokines mainly enhance the activity of macrophages to kill bacteria, while Th2 cytokines inhibit the release of Th1 cytokines, thereby weakening the immune response. The imbalance of Th1/Th2 is directly related to the occurrence and development of TB (77). As previously described, both MSCs and MSCs-EVs have been shown to regulate Th1/Th2 homeostasis, and whether MSCs have the same effect in the Mtb infection microenvironment needs to be further confirmed.

Neutrophils are the most abundant cell type in the innate immune system. They target pathogens through phagocytosis, release of bactericidal molecules and so-called neutrophil extracellular traps, and they are key promoters of antibacterial response (78). During Mtb infection, neutrophil inflammation may be related to lung pathology and TB progression (79, 80). However, neutrophils have a short lifespan. Strategies for enhancing the lifespan and activities of neutrophil can be useful in TB. It has been found that MSCs can recruit neutrophils and extend their life span and pro-inflammatory activity (81). The results of a murine sepsis model showed that MSCs injection assisted bacterial clearance by enhancing the phagocytic activity of neutrophils (82).

Macrophages are the main host cells of Mtb in the body and they are also important cells that constitute TG. It has been found that MSCs and MSCs-EVs can regulate the polarization of M1-type macrophages to M2 (83). And the activation state or polarization of macrophages is crucial to host immune response to pathogen invasion (84). It is reported that the M2 macrophages were found to predominate in both necrotizing and non-necrotizing granulomas of TB patients. In addition, M1 macrophages have high bactericidal activity *in vitro* and promote the formation of granulomas, while M2 macrophages inhibit these effects and play a leading role in the formation of advanced granulomas (85). MSCs reprogram host macrophages in a PGE2-dependent manner to significantly reduce bacteremia and mortality in sepsis (86). Host lipids are an important energy source for Mtb and a safe place for bacteria. Mtb infection can induce the accumulation of cholesterol esters and glycerides in macrophages, leading to the formation of foamy macrophages and TG. Studies have shown that foam macrophages containing LDs promote the persistence of Mtb (87). Furthermore, Mtb-induced LDs formation attenuates host cellular microbicidal activities, thus creating a niche that is ideally suited to its persistent lifestyle (1). Zhang et al. found that MSCs can regulate the expression of scavenger receptor (SR) such as CD36, SRA1 and ABCA1, thereby affecting the lipid homeostasis of macrophages and inhibiting the formation of foam cells. In addition, MSCs can reduce the expression of MHCII in macrophages, thereby reducing the uptake of oxidative low-density lipoprotein (66) (**Figure 2E**). Therefore, MSCs may block Mtb’s energy source for survival and reproduction by regulating the lipid metabolism of macrophages.

#### Regulate Cell Survival

Mtb can infect alveolar epithelial cells, which may provide a protected intracellular environment that is conducive to bacterial replication (88). However, recent work has shown that the Mtb pathogen phosphatidylinositol mannoside may increase the production of reactive oxygen species (ROS) and TGF-β to induce alveolar epithelial cell apoptosis and promote the spread of Mtb (89). Interestingly, keratinocyte growth factor (KGF) and hepatocyte growth factor (HGF) secreted by MSCs protect alveolar epithelial cells from apoptosis under hypoxic conditions by stabilization of endogenous Bcl-2, inhibition of HIF-1α expression and ROS production (90) (**Figure 2F**). Whether MSCs can reduce the transmission of Mtb by inhibiting the apoptosis of alveolar epithelial cells remains to be further studied.

#### Antibacterial Action

MSCs display further disease limiting capacities. Although it has been studied that MSCs can restrict the growth of Mtb by secreting NO, whether MSCs can affect the growth of Mtb through other ways still needs further study (**Figure 2H**). Antimicrobial peptides are an important part of the body’s natural immune system, and they have anti-microbial activity (91). MSCs exhibit direct or indirect antimicrobial activity against a variety of bacterial pathogens, including *Escherichia coli*, *Pseudomonas aeruginosa*, *Staphylococcus aureus*, and *Streptococcus pneumoniae*, by secreting antimicrobial peptides and inducing host innate responses (92–94). It is not known whether MSCs affect the growth of Mtb in the same way.

Autophagy occurs at the basal level of all eukaryotic cells and maintains bioenergy homeostasis by controlling molecular degradation and organelle renewal (95). It is also an emerging innate mechanism of phagocytes. Mtb infection can induce autophagy in phagocytes, thus reducing the viability of intracellular mycobacterium (56, 57, 96). Moreover, autophagy seems to be an inherent feature of many types of stem cells and is closely related to self-renewal, pluripotency and differentiation (97). It was found that MSCs could also hamper the growth of intracellular Mtb through autophagy (25). An *in vitro* study showed that rapamycin can reduce the load of dormant Mtb in MSCs by inducing autophagy (22). In addition, Cen et al. found that rapamycin can activate the autophagy of MSCs and then regulate the recruitment of CD4^+^T cells by MSCs through affecting CXCL8 and TGF-β1, effectively alleviating the inflammatory response (98). However, the autophagy of MSCs may contribute to the spread of Mtb. Granuloma is a hypoxic microenvironment (99). MSCs have a strong role in promoting angiogenesis in hypoxic microenvironment (100). Although angiogenesis can benefit the host by providing a direct source for the arrival of immune system cells against the pathogen, it also facilitates Mtb growth and spread to other tissues (46). Research found that rapamycin-induced autophagy caused MSCs to secrete higher levels of vascular endothelial growth factor (VEGF), which in turn promoted angiogenesis (101). Whether the autophagy of MSCs is an “assist” of Mtb dissemination or a “good medicine” to inhibit the growth of Mtb still needs to be further confirmed.

#### Repairing Lung Damage

TB is a destructive process that leads to scarring of the lungs, parenchymal changes and bronchiectasis, resulting in reduced lung volumes and effects on pulmonary function (102). In addition, studies have shown that there is lung damage after TB treatment (103, 104), and increases risk for airflow obstruction and chronic obstructive pulmonary disease (COPD) (105, 106). And in the preclinical model of Bronchopulmonary dysplasia, MSCs was effective in cytokine accumulation and by improving alveolar and vascular lung structure and function (107, 108). MSCs enhance autophagy of pulmonary microvascular endothelial cells through PI3K/Akt signal transduction, thereby reducing the severity of lung injury caused by ischemia/reperfusion (109) (**Figure 2G**). And in a mouse model of E. coli pneumonia, the activation of toll-like receptor 4 (TLR4) on mouse MSCs enhanced lipocalin-2 activity, improved survival, reduced lung injury, and promotes bacterial clearance (110, 111) (**Figure 2H**). Furthermore, MSCs can attenuate the severity of Escherichia coli-induced acute lung injury, decrease physiologic indices of lung dysfunction, and reduce structural lung injury (112). Besides, MSCs-EVs reduce lung inflammation and protein permeability therefore preventing the formation of pulmonary oedema (113). In the model of silica induced lung injury in mice, MSCs-EVs were able reduce the size of silicotic nodules (114). They also reduce the accumulation of neutrophils and lymphocytes in bronchial lavage fluid and the expression of pulmonary inflammation and pro-fibrosis genes.

MSCs can also be used to treat pulmonary fibrosis (PF). PF is a chronic lung disease in which fibrous granuloma tissue is formed during the progression. Patients with idiopathic pulmonary fibrosis (IPF) are at higher risk of developing TB, Cao et al. have shown that pulmonary resistant mesenchymal stem cells (LR-MSCs) can be activated and differentiated by Wnt/β-catenin signal and participate in the progression of pulmonary fibrosis (115). In another study using MSCs to treat pneumonia, it was found that early intervention of MSCs reduced inflammation and slowed down the process of PF (116). Due to the reduction of pulmonary interstitial inflammatory cells after MSCs infusion, there is less collagen fiber deposition in the lung tissue (116). Therefore, the damage to the lung structure is correspondingly reduced. Furthermore, MSCs can also reduce the degree of fibrosis in the lungs by reducing levels of hydroxy proline, collagen type 1 (COL-1) and COL-3, as well as by paracrine (117).

#### Applications of MSCs Therapy

**Figure 3:**
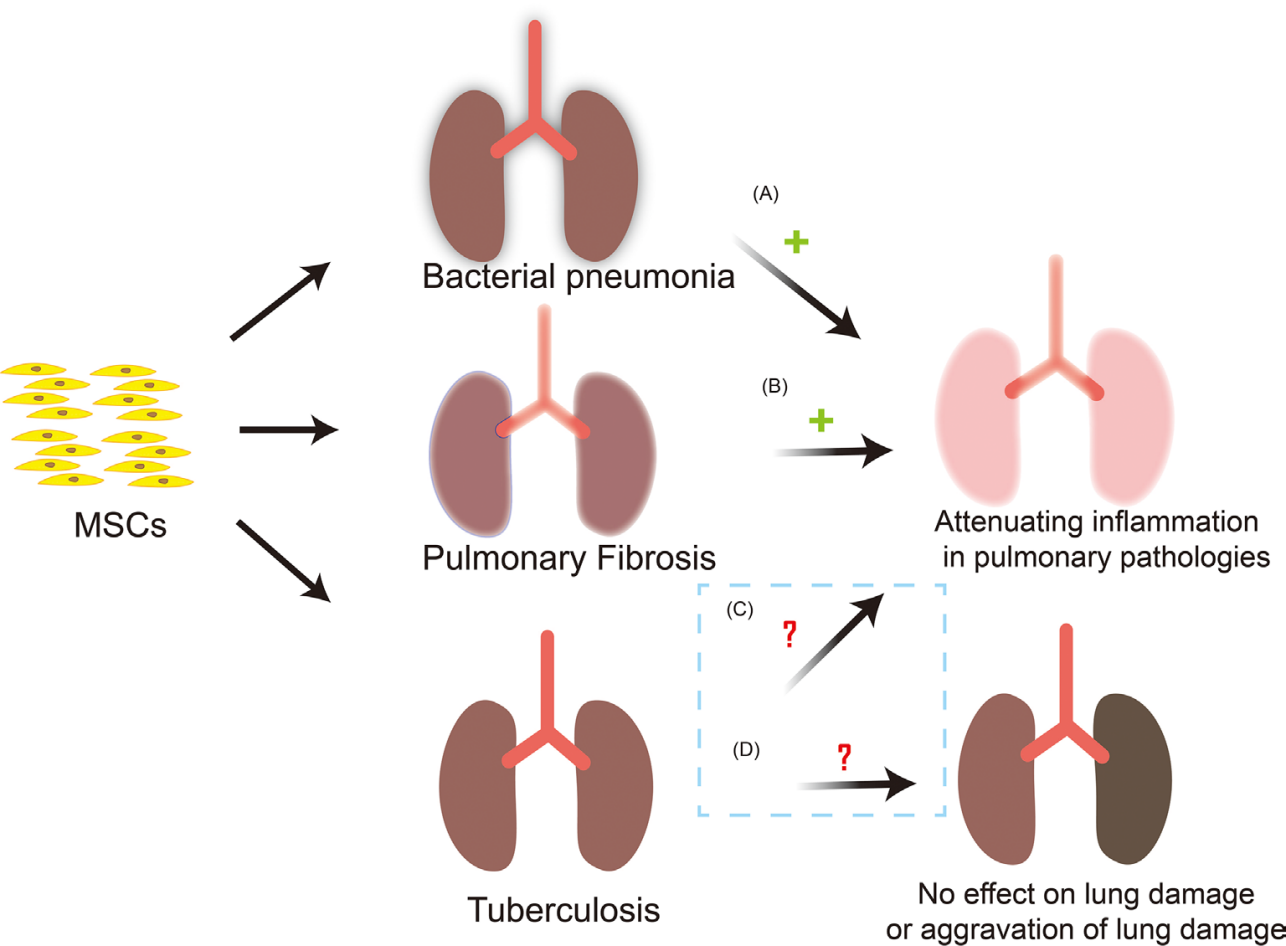
MSCs-based clinical application and possible outcomes for TB. MSCs are used in the treatment of bacterial pneumonia and PF, and have achieved good results. Nevertheless, the safety and effectiveness of MSCs in the treatment of tuberculosis need to be further confirmed. **(A)** MSCs can reduce the severity of bacterial pneumonia, reduce the bacterial load in the lungs and inhibits inflammation, and reduce lung injury. **(B)** After MSCs infusion, there are fewer inflammatory cells in the lung interstitium and collagen fibers. The deposition in lung tissue is reduced and the degree of pulmonary fibrosis is reduced. **(C)** MSCs infusion can effectively reduce the inflammatory response *in vivo*, hinder the development of fibrosis in the lesion site, and does improve the prognosis of TB patients to a large extent. However, the safety and effectiveness need to be verified. **(D)** During Mtb infection, MSCs may not be able to effectively regulate the local immune response and the course of disease. In addition, MSCs have immunosuppressive properties, and its infusion may have the risk of worsening of TB or the reactivation of latent Mtb, so the safety and effectiveness of MSCs in the treatment of TB are issues worthy of in-depth exploration. +: outcomes are effective; ?: outcomes are uncertain.

Lung tissue is the main site of Mtb infection. MSCs has been reported to preferentially accumulate in the lungs following intravenous administration but it is located in the alveolar septa as cell aggregates and does not penetrate into the inflammatory lesions. In addition, during Mtb infection, MSCs cannot significantly regulate the local immune response and disease course of mice, indicating that MSCs cannot effectively treat active TB (118). MSCs infusion may worsen TB or reactivate latent Mtb through immunosuppressive function. However, systemic transplantation of autologous MSCs were found to have a favorable therapeutic effect on 15 patients with MDR-TB and 12 patients with extensive drug resistance TB (XDR-TB) (119). And the results of a phase I trial of autologous BM-MSCs infusion for adjunct treatment of terminally patients with XDR/MDR-TB in Belarus indicated that MSCs infusion may be safe as an adjuvant therapy (120). Then clinical studies showed that 81 percent of patients were treated successfully. Although there are side effects such as hypercholesterolemia and nausea, MSCs therapy does improve the prognosis of MDR-TB to a great extent (121). A model of bladder TB in New Zealand rabbits found that a single-dose administration of MSCs into the bladder mucosal layer significantly reduced the wall deformation and inflammation, and hindered the development of fibrosis, which confirmed the therapeutic efficacy of the interstitial injection of bone-derived MSCs combined with standard anti-TB treatment in the restoration of the bladder function was demonstrated (122). Therefore, MSCs also have good application value in the treatment of extrapulmonary TB. Notably, some reports in the literature indicate that MSCs can trigger rather than inhibit lymphocyte activation when a very low number of MSCs are co-cultured with lymphocytes (123). This means that the ratio between the number of MSCs and immune cells is the key to predict whether MSCs will inhibit or activate the immune system. Consequently, the appropriate dosage for treatment with MSCs may be the key to treatment. In general, *in vivo* results are encouraging, but the safety and effectiveness of MSCs in treating TB remains to be further confirmed due to the lack of a large number of clinical data (**Figure 3**). Through searching the Pubmed and ClinicalTrials.gov database, 4 MSCs-based clinical application for TB were identified which were described in detail in the **Table 2**.

**Table 2:** MSCs-Based Clinical Application for TB.

Type of Cells	Actual Enrollment	Curative effect	Adverse events	Phase	Country	Status	Clinical trials identifier	References
MSCs	Spinal TB(n=20)	unknown	–	Phase 2	Indonesia	Recruiting	NCT04493918(USA)	https://clinicaltrials.gov
Autologous MSCs	MDR-TB(n=15)XDR-TB(n=12)	positive	–	–	Russia	Completed	–	(119),
Autologous BM-MSCs	MDR/XDR-TB(n=30)	positive	high cholesterol levels (14/30, nausea (11/30) lymphopenia or diarrhea (10/30)	phase I	Belarus	Completed	DRKS00000763 (German)	(120),
Autologous BM-MSCs	MDR/XDR-TB(n=36)	positive	hypercholesterolaemia and nausea	–	Belarus	Completed	–	(121)

MSCs, mesenchymal stem cells; BM-MSCs, bone marrow mesenchymal stem cells; MDR-TB, multidrug-resistant tuberculosis; XDR-TB, extensive drug resistance tuberculosis.

#### Unanswered Questions and Future Perspectives of MSCs for TB

At present, MSCs are the hot point cells in the medical field in recent years, which have a good application prospect in the treatment of inflammatory or immune-related diseases and regenerative medicine. Although several clinical studies of tuberculosis treatment based on MSCs have shown positive results, the research on the relationship between MSCs and TB is still in the exploratory stage. Therefore, MSCs-based TB treatment should pay attention to the following issues. 1). There were some differences in the biological functions of MSCs in different tissue sources and different culture conditions *in vitro* (124, 125). It is very important to select MSCs from appropriate tissue sources and optimize the culture conditions *in vitro* to obtain high-quality and efficient MSCs. 2). At present, MSCs treatment still has some side effects (126). Although MSCs are safe as an adjuvant therapy for TB treatment, it cannot be fully proved due to the lack of extensive clinical trial data. 3). Studies have found that TB has different symptoms, different microbial composition, different immune responses and pathological characteristics, and there are multiple forms of infection even in a single individual (127). Therefore, treatment should be carefully selected according to individual immune status. In addition, various factors should be considered when using MSCs for treatment, such as the stage of the disease, related HIV infection or other conditions, including genetic susceptibility, and indications for MSCs infusion. 4). Mtb can infect macrophages, alveolar epithelial cells and MSCs. However, there are differences in the lifestyle of Mtb in host cells, which may be one of the reasons for Mtb resistance. Therefore, fully understanding the relationship between Mtb and host cells is the key to the treatment of TB. 5). MSCs-EVs also have immunomodulatory and pluripotency (128), and EVs have relatively single structure and small volume, which may have stronger permeability and safety (129). However, MSCs-EVs could act *via* different mechanisms from that of their progenitor MSCs (130). Therefore, MSCs-EVs may be a potential treatment tool for TB.

### Application Value of MSCs in TB Model

The current research on the pathogenesis of TB is limited to the representative human TG model. The method of establishing TG model is very limited, mainly relying on animal models. The most common laboratory animals are mice, guinea, pigs and zebrafish. However, the dormancy properties of Mtb in humans cannot be generalized in any available animal model. So a new system is needed to study and understand the natural processes of infection and disease progression (131). In addition, TG in non-human primates is most similar to human TG but cannot be widely used due to high operating costs and ethical issues.

In recent years, many scientists have established human TG models *in vitro* for TB research (132–134). Although these models are physiologically relevant to human TB and are less costly and have no ethical concerns than animal models, they have some limitations, such as the inability to simulate the chronic course of TB. Interestingly, *in vitro* studies have shown that Mtb can survive long-term in a non-proliferative state in MSCs without affecting the viability of host cells at low levels of infection (26). In addition, Mtb gradually increases resistance to antibiotics, a phenotype associated with dormant bacilli (26). However, infection of the human macrophage THP-1 showed a time-dependent increase in Mtb burden while also causing host cell death, even at a low MOI of 1:1 (26). In addition, it is speculated that the invasion and replication of Mtb in alveolar epithelial cells may overwhelm them, leading to cell death and bacterial dissemination (135). Therefore, compared with the commonly used *in vitro* TB models of macrophages and alveolar epithelial cells, MSCs can better reflect the incubation period of Mtb and simulate the chronic course of TB. Unfortunately, the existing *in vitro* models do not include MSCs.

The granulomatous response is considered to be a necessary step for the anti-TB response. A good vaccine candidate must be a good granuloma inducer (136). As we have mentioned before, MSCs may be candidates for TB vaccines due to their strong immunoregulatory ability and may be involved in the formation of TG. It is important to note that although the BCG vaccine showed good granulomatous induction, it did not protect 100% of the immunized individuals against TB (136). Therefore, inducing TG formation may not be a sufficient condition for inducing protection, but it can be used as a screening index for TB vaccine. Therefore, our research team is currently using *in vitro* models to study the ability of MSCs and existing vaccines to promote the formation of TG, which may help to develop alternative vaccines for TB.

### Potential of MSCs to Diagnose

Pulmonary tuberculosis (PTB) can be diagnosed with clinical symptoms (chronic cough, sputum expectoration, low fever, night sweats, hemoptysis, etc.), pulmonary CT, skin test, and sputum smears. However, the diagnosis of latent tuberculosis infection (LTBI) is fairly indirect relying on evidence of cellular immune response to mycobacterial antigens (137). The most commonly used tests for LTBI diagnosis are the introcyte tuberculosis mycosin test (TST) and IFN-γ release assays (IGRA) (138, 139). The IGRA is based on the detection of lymphocytes stimulated by a peptide specifically encoded by Mtb diagnostic test for *in vitro* secretion of IFN-γ. However, IGRA is based on the quantification of IFN-γ after only brief lymphocyte stimulation (16-24 hours) but may not be enough to cause the release of markers from central memory T cells (140). In addition, an important proportion of subjects with advanced PTB can be insulating to immune stimulation and IGRAs can also lead to false negative or indeterminate results, so neither TST nor IGRA can be used to distinguish between PTB and LTBI (141). Granulocyte-macrophage colony-stimulating factor (GM-CSF) and IL-2 were more active than IGRA reported at the highest specificity, distinguish PTB from LTBI and uninfected TB (142). In addition, these tests cannot be used to predict whether patients with LTBI will develop PTB, and treatment of LTBI cannot effectively reduce the risk of PTB.

Recently, the frequency of CD57-expressing cells may provide another way to distinguish between the two subjects. CD57-expressing cells are more frequently expressed in PTB patients than LTBI, and can be used to identify patients with active disease prone to LTBI infection. Studies have shown that the frequency of CD57-expressing cells may provide another way to distinguish these two subjects. The expression frequency of CD57-expressing cells in PTB patients is higher than that of LTBI, and can be used to identify LTBI infection patients who are prone to develop active diseases (143). In addition, compared with LTBI, patients with extrapulmonary tuberculosis (EPTB) and PTB have a higher frequency of CD4^+^ T cells expressing CD38+, HLADR or Ki67 (P <0.001), which can separate active TB from LTBI and EPTB from PTB (144). These parameters provide an attractive way to develop blood-based diagnostic tests for PTB and LTBI. And T-cell enzyme-linked immune speckle method (T-SPOT^®^. TB) is highly sensitive and specific to TB and PTB, and is not affected by previous TB or BCG vaccination history (145). However, patients with poor immunity, nutritional status, and decreased T cell quantity and function in the elderly may reduce the sensitivity of T-SPOT^®^.TB. In addition, *Escherichia coli*, *Mycobacterium kansasii*, or *Mycobacterium gordonae *infection can cause false positives in experimental results (145). A recent study found that large volumes are required to get adequate cerebrospinal fluid mononuclear cells when the assay is used on cerebrospinal fluid, which hinders its clinical use (146). Therefore, the specificity and sensitivity of T-SPOT^®^ needs to be further improved. Notably, Banerjee et al. found that MSCs surface marker CD73 was of reference value in the differential diagnosis of intestinal TG and granuloma in Crohn’s disease (147, 148). Crohn’s disease is an inflammatory bowel disease caused by dysregulation of immune tolerance in genetically susceptible individuals (149), it presents as chronic granulomatous disorder but is not significantly associated with pathogen infection. And it is tempting to suggest that CD73^+^MSCs are involved in TG formation and may have Mtb or bacterial specificity. In addition, MSCs can regulate T cell function, and the addition of MSCs to the T-SPOT^®^. TB system may amplify the antigen-specific T cell effect and enhance the sensitivity and specificity of the assay.

## Conclusion

As a new class of phagocytes and immune cells, MSCs have been found to exist in TG, which can not only provide a drug-tolerant and immune-privileged niche for Mtb dormant, but also have the ability to restrict the growth of Mtb to a certain extent and may be involved in the occurrence and development of TB. In addition, dormant Mtb induced quiescence in MSCs and promoted their long-term survival. Like macrophages, DCs, classical T cells and non-classical immune cells, MSCs also have strong immunomodulatory/anti-inflammatory, antibacterial, repair of damaged tissue and other abilities during the period involved in regulating TB immune responses, so they can not only be used as an immune target or immunotherapy agent for the treatment of TB, but also may provide a screening model for the development of new drugs or vaccines for TB. In summary, we believed that with further in-depth research on the relationship between MSCs and TB, it will surely bring good news to TB patients worldwide.

## Author Contributions

XZ, QX, ZY, YL, and ZC performed the relevant literature search and wrote the review manuscript. MH and JZ reviewed the manuscript for submission. All authors contributed to the article and approved the submitted version.

## Funding

This study was supported by grants from the Natural Science Foundation of Guangdong Province (2019A1515011713), Characteristic Innovation Experimental Project of Ordinary Universities in Guangdong Province (2020KTSCX044), Discipline Construction Project of Guangdong Medical University (4SG21266P), Research Foundation of Guangdong Medical University for Ph.D. Staff (GDMUB2019038, GDMUB2020017), the Medical Science Foundation of Guangdong Province (A2021438).

## Conflict of Interest

The authors declare that the research was conducted in the absence of any commercial or financial relationships that could be construed as a potential conflict of interest.

## References

[1] Singh V Jamwal S Jain R Verma P Gokhale R Rao KV. Mycobacterium Tuberculosis-Driven Targeted Recalibration of Macrophage Lipid Homeostasis Promotes the Foamy Phenotype. Cell Host Microbe (2012) 12:669–81. 10.1016/j.chom.2012.09.012 23159056

[2] Churchyard G Kim P Shah NS Rustomjee R Gandhi N Mathema B. What We Know About Tuberculosis Transmission: An Overview. J Infect Dis (2017) 216:S629–S35. 10.1093/infdis/jix362 PMC5791742 29112747

[3] Cohen SB Gern BH Delahaye JL Adams KN Plumlee CR Winkler JK. Alveolar Macrophages Provide an Early Mycobacterium Tuberculosis Niche and Initiate Dissemination. Cell Host Microbe (2018) 24:439–46 e4. 10.1016/j.chom.2018.08.001 30146391 PMC6152889

[4] Orme IM Basaraba RJ. The Formation of the Granuloma in Tuberculosis Infection. Semin Immunol (2014) 26:601–9. 10.1016/j.smim.2014.09.009 25453231

[5] Moreno V Espinoza B Barley K Paredes M Bichara D Mubayi A. The Role of Mobility and Health Disparities on the Transmission Dynamics of Tuberculosis. Theor Biol Med Model (2017) 14:3. 10.1186/s12976-017-0049-6 28129769 PMC5273827

[6] Kothari H Keshava S Vatsyayan R Mackman N Rao LV Pendurthi UR. Role of Tissue Factor in Mycobacterium Tuberculosis-Induced Inflammation and Disease Pathogenesis. PloS One (2014) 9:e114141. 10.1371/journal.pone.0114141 25462128 PMC4252100

[7] Sandor M Weinstock JV Wynn TA. Granulomas in Schistosome and Mycobacterial Infections: A Model of Local Immune Responses. Trends Immunol (2003) 24:44–52. 10.1016/s1471-4906(02)00006-6 12495724

[8] Tufariello JM Chan J Flynn JL. Latent Tuberculosis: Mechanisms of Host and Bacillus That Contribute to Persistent Infection. Lancet Infect Dis (2003) 3:578–90. 10.1016/s1473-3099(03)00741-2 12954564

[9] Dominici M Le Blanc K Mueller I Slaper-Cortenbach I Marini F Krause D. Minimal Criteria for Defining Multipotent Mesenchymal Stromal Cells. The International Society for Cellular Therapy Position Statement. Cytotherapy (2006) 8:315–7. 10.1080/14653240600855905 16923606

[10] Zhang Q Shi S Liu Y Uyanne J Shi Y Shi S. Mesenchymal Stem Cells Derived From Human Gingiva Are Capable of Immunomodulatory Functions and Ameliorate Inflammation-Related Tissue Destruction in Experimental Colitis. J Immunol (2009) 183:7787–98. 10.4049/jimmunol.0902318 PMC2881945 19923445

[11] Zhang Q Nguyen P Xu Q Park W Lee S Furuhashi A. Neural Progenitor-Like Cells Induced From Human Gingiva-Derived Mesenchymal Stem Cells Regulate Myelination of Schwann Cells in Rat Sciatic Nerve Regeneration. Stem Cells Transl Med (2017) 6:458–70. 10.5966/sctm.2016-0177 PMC5442816 28191764

[12] Xiang B Chen L Wang X Zhao Y Wang Y Xiang C. Transplantation of Menstrual Blood-Derived Mesenchymal Stem Cells Promotes the Repair of LPS-Induced Acute Lung Injury. Int J Mol Sci (2017) 18:689. 10.3390/ijms18040689 PMC5412275 28346367

[13] Sala E Genua M Petti L Anselmo A Arena V Cibella J. Mesenchymal Stem Cells Reduce Colitis in Mice via Release of TSG6, Independently of Their Localization to the Intestine. Gastroenterology (2015) 149:163–76.e20. 10.1053/j.gastro.2015.03.013 25790743

[14] Chen M Peng J Xie Q Xiao N Su X Mei H. Mesenchymal Stem Cells Alleviate Moderate-To-Severe Psoriasis by Reducing the Production of Type I Interferon (IFN-I) by Plasmacytoid Dendritic Cells (pDCs). Stem Cells Int (2019) 2019:6961052. 10.1155/2019/6961052 31827531 PMC6885248

[15] Liu X Wang Z Song W Sun W Hong R Pothukuchi A. Systematically Transplanted Human Gingiva-Derived Mesenchymal Stem Cells Regulate Lipid Metabolism and Inflammation in Hyperlipidemic Mice With Periodontitis. Exp Ther Med (2020) 19:672–82. 10.3892/etm.2019.8256 PMC6913381 31885706

[16] Ramakrishnan L. Revisiting the Role of the Granuloma in Tuberculosis. Nat Rev Immunol (2012) 12:352–66. 10.1038/nri3211 22517424

[17] Raghuvanshi S Sharma P Singh S Van Kaer L Das G. Mycobacterium Tuberculosis Evades Host Immunity by Recruiting Mesenchymal Stem Cells. Proc Natl Acad Sci USA (2010) 107:21653–8. 10.1073/pnas.1007967107 PMC3003090 21135221

[18] Das B Kashino SS Pulu I Kalita D Swami V Yeger H. CD271(+) Bone Marrow Mesenchymal Stem Cells May Provide a Niche for Dormant Mycobacterium Tuberculosis. Sci Transl Med (2013) 5:170ra13. 10.1126/scitranslmed.3004912 PMC3616630 23363977

[19] Rustad TR Sherrid AM Minch KJ Sherman DR. Hypoxia: A Window Into Mycobacterium Tuberculosis Latency. Cell Microbiol (2009) 11:1151–9. 10.1111/j.1462-5822.2009.01325.x 19388905

[20] Garhyan J Bhuyan S Pulu I Kalita D Das B Bhatnagar R. Preclinical and Clinical Evidence of Mycobacterium Tuberculosis Persistence in the Hypoxic Niche of Bone Marrow Mesenchymal Stem Cells After Therapy. Am J Pathol (2015) 185:1924–34. 10.1016/j.ajpath.2015.03.028 26066709

[21] Iso Y Yamaya S Sato T Poole CN Isoyama K Mimura M. Distinct Mobilization of Circulating CD271+ Mesenchymal Progenitors From Hematopoietic Progenitors During Aging and After Myocardial Infarction. Stem Cells Transl Med (2012) 1:462–8. 10.5966/sctm.2011-0051 PMC3659716 23197850

[22] Fatima S Kamble SS Dwivedi VP Bhattacharya D Kumar S Ranganathan A. Mycobacterium Tuberculosis Programs Mesenchymal Stem Cells to Establish Dormancy and Persistence. J Clin Invest (2020) 130:655–61. 10.1172/JCI128043 PMC6994115 31647784

[23] Jain N Kalam H Singh L Sharma V Kedia S Das P. Mesenchymal Stem Cells Offer a Drug-Tolerant and Immune-Privileged Niche to Mycobacterium Tuberculosis. Nat Commun (2020) 11:3062. 10.1038/s41467-020-16877-3 32546788 PMC7297998

[24] Schwartz YS Belogorodtsev SN Filimonov PN Cherednichenko AG Pustylnikov SV Krasnov VA. BCG Infection in Mice Is Promoted by Naive Mesenchymal Stromal Cells (MSC) and Suppressed by Poly(A:U)-Conditioned MSC. Tuberculosis (Edinb) (2016) 101:130–36. 10.1016/j.tube.2016.09.005 27865382

[25] Khan A Mann L Papanna R Lyu MA Singh CR Olson S. Mesenchymal Stem Cells Internalize Mycobacterium Tuberculosis Through Scavenger Receptors and Restrict Bacterial Growth Through Autophagy. Sci Rep (2017) 7:15010. 10.1038/s41598-017-15290-z 29118429 PMC5678154

[26] Singh VK Mishra A Bark S Mani A Subbian S Hunter RL. Human Mesenchymal Stem Cell Based Intracellular Dormancy Model of Mycobacterium Tuberculosis. Microbes Infect (2020) 22:423–31. 10.1016/j.micinf.2020.05.015 PMC8059136 32562667

[27] Pal B Das B. In Vitro Culture of Naive Human Bone Marrow Mesenchymal Stem Cells: A Stemness Based Approach. Front Cell Dev Biol (2017) 5:69. 10.3389/fcell.2017.00069 28884113 PMC5572382

[28] Pathak L Das B. Initiation of Post-Primary Tuberculosis of the Lungs: Exploring the Secret Role of Bone Marrow Derived Stem Cells. Front Immunol (2020) 11:594572. 10.3389/fimmu.2020.594572 33584661 PMC7873989

[29] Das B Pathak L Gayan S Pal B Saikia P Baishya T. Stem Cell Basis of a Host Driven Transmission of Antigen Packed Aerosols: A Novel Mechanism of Natural Vaccination for Tuberculosis. bioRxiv (2020), 382572. 10.1101/2020.11.14.382572

[30] Zhen G Wen C Jia X Li Y Crane JL Mears SC. Inhibition of TGF-Beta Signaling in Mesenchymal Stem Cells of Subchondral Bone Attenuates Osteoarthritis. Nat Med (2013) 19:704–12. 10.1038/nm.3143 PMC3676689 23685840

[31] Coppin L Sokal E Stephenne X. Thrombogenic Risk Induced by Intravascular Mesenchymal Stem Cell Therapy: Current Status and Future Perspectives. Cells (2019) 8:1160. 10.3390/cells8101160 PMC6829440 31569696

[32] Caccamo N Dieli F. Inflammation and the Coagulation System in Tuberculosis: Tissue Factor Leads the Dance. Eur J Immunol (2016) 46:303–6. 10.1002/eji.201546225 26763085

[33] Gleeson BM Martin K Ali MT Kumar AH Pillai MG Kumar SP. Bone Marrow-Derived Mesenchymal Stem Cells Have Innate Procoagulant Activity and Cause Microvascular Obstruction Following Intracoronary Delivery: Amelioration by Antithrombin Therapy. Stem Cells (2015) 33:2726–37. 10.1002/stem.2050 25969127

[34] Munir H Ward LSC Sheriff L Kemble S Nayar S Barone F. Adipogenic Differentiation of Mesenchymal Stem Cells Alters Their Immunomodulatory Properties in a Tissue-Specific Manner. Stem Cells (2017) 35:1636–46. 10.1002/stem.2622 PMC6052434 28376564

[35] Chen JX Han YS Zhang SQ Li ZB Chen J Yi WJ. Novel Therapeutic Evaluation Biomarkers of Lipid Metabolism Targets in Uncomplicated Pulmonary Tuberculosis Patients. Signal Transduct Target Ther (2021) 6:22. 10.1038/s41392-020-00427-w 33462176 PMC7814055

[36] Zhang C Baker DL Yasuda S Makarova N Balazs L Johnson LR. Lysophosphatidic Acid Induces Neointima Formation Through PPARgamma Activation. J Exp Med (2004) 199:763–74. 10.1084/jem.20031619 PMC2212723 15007093

[37] Almeida PE Carneiro AB Silva AR Bozza PT. PPARgamma Expression and Function in Mycobacterial Infection: Roles in Lipid Metabolism, Immunity, and Bacterial Killing. PPAR Res (2012) 2012:383829. 10.1155/2012/383829 22851964 PMC3407650

[38] Almeida PE Silva AR Maya-Monteiro CM Torocsik D D’Avila H Dezso B. Mycobacterium Bovis Bacillus Calmette-Guerin Infection Induces TLR2-Dependent Peroxisome Proliferator-Activated Receptor Gamma Expression and Activation: Functions in Inflammation, Lipid Metabolism, and Pathogenesis. J Immunol (2009) 183:1337–45. 10.4049/jimmunol.0900365 19561094

[39] Li Y Jin D Xie W Wen L Chen W Xu J. PPAR-Gamma and Wnt Regulate the Differentiation of MSCs Into Adipocytes and Osteoblasts Respectively. Curr Stem Cell Res Ther (2018) 13:185–92. 10.2174/1574888X12666171012141908 29034841

[40] Beigier-Bompadre M Montagna GN Kuhl AA Lozza L Weiner J 3rd Kupz A. Mycobacterium Tuberculosis Infection Modulates Adipose Tissue Biology. PloS Pathog (2017) 13:e1006676. 10.1371/journal.ppat.1006676 29040326 PMC5695609

[41] Champion TC Partridge LJ Ong SM Malleret B Wong SC Monk PN. Monocyte Subsets Have Distinct Patterns of Tetraspanin Expression and Different Capacities to Form Multinucleate Giant Cells. Front Immunol (2018) 9:1247. 10.3389/fimmu.2018.01247 29937768 PMC6002745

[42] Krishnan D Ghosh SK. Cellular Events of Multinucleated Giant Cells Formation During the Encystation of Entamoeba Invadens. Front Cell Infect Microbiol (2018) 8:262. 10.3389/fcimb.2018.00262 30109218 PMC6079502

[43] Miron RJ Bosshardt DD. Multinucleated Giant Cells: Good Guys or Bad Guys? Tissue Eng Part B Rev (2018) 24:53–65. 10.1089/ten.TEB.2017.0242 28825357

[44] Braid LR Wood CA Wiese DM Ford BN. Intramuscular Administration Potentiates Extended Dwell Time of Mesenchymal Stromal Cells Compared to Other Routes. Cytotherapy (2018) 20:232–44. 10.1016/j.jcyt.2017.09.013 29167063

[45] Jungwirth N Salinas Tejedor L Jin W Gudi V Skripuletz T Stein VM. Mesenchymal Stem Cells Form 3D Clusters Following Intraventricular Transplantation. J Mol Neurosci (2018) 65:60–73. 10.1007/s12031-018-1070-x 29705933

[46] Batista LAF Silva KJS da Costa ESLM de Moura YF Zucchi FCR. Tuberculosis: A Granulomatous Disease Mediated by Epigenetic Factors. Tuberculosis (Edinb) (2020) 123:101943. 10.1016/j.tube.2020.101943 32741528

[47] Bartosh TJ Ylostalo JH Mohammadipoor A Bazhanov N Coble K Claypool K. Aggregation of Human Mesenchymal Stromal Cells (MSCs) Into 3D Spheroids Enhances Their Antiinflammatory Properties. Proc Natl Acad Sci USA (2010) 107:13724–9. 10.1073/pnas.1008117107 PMC2922230 20643923

[48] Lin PL Flynn JL. CD8 T Cells and Mycobacterium Tuberculosis Infection. Semin Immunopathol (2015) 37:239–49. 10.1007/s00281-015-0490-8 PMC4439333 25917388

[49] Bertholet S Ireton GC Kahn M Guderian J Mohamath R Stride N. Identification of Human T Cell Antigens for the Development of Vaccines Against Mycobacterium Tuberculosis. J Immunol (2008) 181:7948–57. 10.4049/jimmunol.181.11.7948 PMC2586986 19017986

[50] Lewinsohn DA Lewinsohn DM Scriba TJ. Polyfunctional CD4(+) T Cells As Targets for Tuberculosis Vaccination. Front Immunol (2017) 8:1262. 10.3389/fimmu.2017.01262 29051764 PMC5633696

[51] Ritz N Hanekom WA Robins-Browne R Britton WJ Curtis N. Influence of BCG Vaccine Strain on the Immune Response and Protection Against Tuberculosis. FEMS Microbiol Rev (2008) 32:821–41. 10.1111/j.1574-6976.2008.00118.x 18616602

[52] Fujimoto T Parton RG. Not Just Fat: The Structure and Function of the Lipid Droplet. Cold Spring Harb Perspect Biol (2011) 3:a004838. 10.1101/cshperspect.a004838 21421923 PMC3039932

[53] Knight M Braverman J Asfaha K Gronert K Stanley S. Lipid Droplet Formation in Mycobacterium Tuberculosis Infected Macrophages Requires IFN-Gamma/HIF-1alpha Signaling and Supports Host Defense. PloS Pathog (2018) 14:e1006874. 10.1371/journal.ppat.1006874 29370315 PMC5800697

[54] Matta SK Kumar D. AKT Mediated Glycolytic Shift Regulates Autophagy in Classically Activated Macrophages. Int J Biochem Cell Biol (2015) 66:121-33. 10.1016/j.biocel.2015.07.010 26222186

[55] Schlesinger LS. Macrophage Phagocytosis of Virulent But Not Attenuated Strains of Mycobacterium Tuberculosis Is Mediated by Mannose Receptors in Addition to Complement Receptors. J Immunol (1993) 150:2920-30. 8454864

[56] Deretic V Singh S Master S Harris J Roberts E Kyei G. Mycobacterium Tuberculosis Inhibition of Phagolysosome Biogenesis and Autophagy as a Host Defence Mechanism. Cell Microbiol (2006) 8:719–27. 10.1111/j.1462-5822.2006.00705.x 16611222

[57] Jagannath C Lindsey DR Dhandayuthapani S Xu Y Hunter RL Jr Eissa NT. Autophagy Enhances the Efficacy of BCG Vaccine by Increasing Peptide Presentation in Mouse Dendritic Cells. Nat Med (2009) 15:267–76. 10.1038/nm.1928 19252503

[58] Liu PT Modlin RL. Human Macrophage Host Defense Against Mycobacterium Tuberculosis. Curr Opin Immunol (2008) 20:371-6. 10.1016/j.coi.2008.05.014 18602003

[59] Szumowski JD Adams KN Edelstein PH Ramakrishnan L. Antimicrobial Efflux Pumps and Mycobacterium Tuberculosis Drug Tolerance: Evolutionary Considerations. Curr Top Microbiol Immunol (2013) 374:81-108. 10.1007/82_2012_300 PMC3859842 23242857

[60] Can Sarinoglu R Sili U Eryuksel E Olgun Yildizeli S Cimsit C Karahasan Yagci A. Tuberculosis and COVID-19: An Overlapping Situation During Pandemic. J Infect Dev Ctries (2020) 14:721–25. 10.3855/jidc.13152 32794460

[61] Lange C Dheda K Chesov D Mandalakas AM Udwadia Z Horsburgh CR Jr. Management of Drug-Resistant Tuberculosis. Lancet (2019) 394:953–66. 10.1016/S0140-6736(19)31882-3 PMC11524526 31526739

[62] Shamputa IC Van Deun A Salim MA Hossain MA Fissette K de Rijk P. Endogenous Reactivation and True Treatment Failure as Causes of Recurrent Tuberculosis in a High Incidence Setting With a Low HIV Infection. Trop Med Int Health (2007) 12:700–8. 10.1111/j.1365-3156.2007.01840.x 17550467

[63] Tsenova L Singhal A. Effects of Host-Directed Therapies on the Pathology of Tuberculosis. J Pathol (2020) 250:636–46. 10.1002/path.5407 32108337

[64] Li P Zhao Y Ge L. Therapeutic Effects of Human Gingiva-Derived Mesenchymal Stromal Cells on Murine Contact Hypersensitivity via Prostaglandin E2-EP3 Signaling. Stem Cell Res Ther (2016) 7:103. 10.1186/s13287-016-0361-9 27484807 PMC4969691

[65] Huang F Chen M Chen W Gu J Yuan J Xue Y. Human Gingiva-Derived Mesenchymal Stem Cells Inhibit Xeno-Graft-Versus-Host Disease via CD39-CD73-Adenosine and IDO Signals. Front Immunol (2017) 8:68. 10.3389/fimmu.2017.00068 28210258 PMC5288353

[66] Zhang X Huang F Li W Dang JL Yuan J Wang J. Human Gingiva-Derived Mesenchymal Stem Cells Modulate Monocytes/Macrophages and Alleviate Atherosclerosis. Front Immunol (2018) 9:878. 10.3389/fimmu.2018.00878 29760701 PMC5937358

[67] Yang R Yu T Liu D Shi S Zhou Y. Hydrogen Sulfide Promotes Immunomodulation of Gingiva-Derived Mesenchymal Stem Cells *via* the Fas/FasL Coupling Pathway. Stem Cell Res Ther (2018) 9:62. 10.1186/s13287-018-0804-6 29523215 PMC5845196

[68] Su WR Zhang QZ Shi SH Nguyen AL Le AD. Human Gingiva-Derived Mesenchymal Stromal Cells Attenuate Contact Hypersensitivity *via* Prostaglandin E2-Dependent Mechanisms. Stem Cells (2011) 29:1849–60. 10.1002/stem.738 21987520

[69] Chao K Zhang S Qiu Y Chen X Zhang X Cai C. Human Umbilical Cord-Derived Mesenchymal Stem Cells Protect Against Experimental Colitis via CD5(+) B Regulatory Cells. Stem Cell Res Ther (2016) 7:109. 10.1186/s13287-016-0376-2 27515534 PMC4981968

[70] Peng Y Chen X Liu Q Zhang X Huang K Liu L. Mesenchymal Stromal Cells Infusions Improve Refractory Chronic Graft Versus Host Disease Through an Increase of CD5+ Regulatory B Cells Producing Interleukin 10. Leukemia (2015) 29:636–46. 10.1038/leu.2014.225 25034146

[71] Yanez-Mo M Siljander PR Andreu Z Zavec AB Borras FE Buzas EI. Biological Properties of Extracellular Vesicles and Their Physiological Functions. J Extracell Vesicles (2015) 4:27066. 10.3402/jev.v4.27066 25979354 PMC4433489

[72] Favaro E Carpanetto A Lamorte S Fusco A Caorsi C Deregibus MC. Human Mesenchymal Stem Cell-Derived Microvesicles Modulate T Cell Response to Islet Antigen Glutamic Acid Decarboxylase in Patients With Type 1 Diabetes. Diabetologia (2014) 57:1664–73. 10.1007/s00125-014-3262-4 24838680

[73] Budoni M Fierabracci A Luciano R Petrini S Di Ciommo V Muraca M. The Immunosuppressive Effect of Mesenchymal Stromal Cells on B Lymphocytes is Mediated by Membrane Vesicles. Cell Transplant (2013) 22:369–79. 10.3727/096368911X582769 23433427

[74] Ren W Hou J Yang C Wang H Wu S Wu Y. Extracellular Vesicles Secreted by Hypoxia Pre-Challenged Mesenchymal Stem Cells Promote non-Small Cell Lung Cancer Cell Growth and Mobility as Well as Macrophage M2 Polarization via miR-21-5p Delivery. J Exp Clin Cancer Res (2019) 38:62. 10.1186/s13046-019-1027-0 30736829 PMC6367822

[75] Reis M Mavin E Nicholson L Green K Dickinson AM Wang XN. Mesenchymal Stromal Cell-Derived Extracellular Vesicles Attenuate Dendritic Cell Maturation and Function. Front Immunol (2018) 9:2538. 10.3389/fimmu.2018.02538 30473695 PMC6237916

[76] Akiyama K Chen C Wang D Xu X Qu C Yamaza T. Mesenchymal-Stem-Cell-Induced Immunoregulation Involves FAS-Ligand-/FAS-Mediated T Cell Apoptosis. Cell Stem Cell (2012) 10:544–55. 10.1016/j.stem.2012.03.007 PMC3348385 22542159

[77] Li Q Zhang H Yu L Wu C Luo X Sun H. Down-Regulation of Notch Signaling Pathway Reverses the Th1/Th2 Imbalance in Tuberculosis Patients. Int Immunopharmacol (2018) 54:24–32. 10.1016/j.intimp.2017.10.026 29100034

[78] Le Blanc K Mougiakakos D. Multipotent Mesenchymal Stromal Cells and the Innate Immune System. Nat Rev Immunol (2012) 12:383–96. 10.1038/nri3209 22531326

[79] Keller C Hoffmann R Lang R Brandau S Hermann C Ehlers S. Genetically Determined Susceptibility to Tuberculosis in Mice Causally Involves Accelerated and Enhanced Recruitment of Granulocytes. Infect Immun (2006) 74:4295–309. 10.1128/IAI.00057-06 PMC1489748 16790804

[80] Lyadova IV Tsiganov EN Kapina MA Shepelkova GS Sosunov VV Radaeva TV. In Mice, Tuberculosis Progression Is Associated With Intensive Inflammatory Response and the Accumulation of Gr-1 Cells in the Lungs. PloS One (2010) 5:e10469. 10.1371/journal.pone.0010469 20454613 PMC2864263

[81] Brandau S Jakob M Hemeda H Bruderek K Janeschik S Bootz F. Tissue-Resident Mesenchymal Stem Cells Attract Peripheral Blood Neutrophils and Enhance Their Inflammatory Activity in Response to Microbial Challenge. J Leukoc Biol (2010) 88:1005–15. 10.1189/jlb.0410207 20682625

[82] Hall SR Tsoyi K Ith B Padera RF Jr. Lederer JA Wang Z. Mesenchymal Stromal Cells Improve Survival During Sepsis in the Absence of Heme Oxygenase-1: The Importance of Neutrophils. Stem Cells (2013) 31:397–407. 10.1002/stem.1270 23132816 PMC3572335

[83] Hyvarinen K Holopainen M Skirdenko V Ruhanen H Lehenkari P Korhonen M. Mesenchymal Stromal Cells and Their Extracellular Vesicles Enhance the Anti-Inflammatory Phenotype of Regulatory Macrophages by Downregulating the Production of Interleukin (IL)-23 and IL-22. Front Immunol (2018) 9:771. 10.3389/fimmu.2018.00771 29706969 PMC5906545

[84] Labonte AC Tosello-Trampont AC Hahn YS. The Role of Macrophage Polarization in Infectious and Inflammatory Diseases. Mol Cells (2014) 37:275–85. 10.14348/molcells.2014.2374 PMC4012075 24625576

[85] Huang Z Luo Q Guo Y Chen J Xiong G Peng Y. Mycobacterium Tuberculosis-Induced Polarization of Human Macrophage Orchestrates the Formation and Development of Tuberculous Granulomas In Vitro. PloS One (2015) 10:e0129744. 10.1371/journal.pone.0129744 26091535 PMC4474964

[86] Nemeth K Leelahavanichkul A Yuen PS Mayer B Parmelee A Doi K. Bone Marrow Stromal Cells Attenuate Sepsis via Prostaglandin E(2)-Dependent Reprogramming of Host Macrophages to Increase Their Interleukin-10 Production. Nat Med (2009) 15:42–9. 10.1038/nm.1905 PMC2706487 19098906

[87] Estrella JL Kan-Sutton C Gong X Rajagopalan M Lewis DE Hunter RL. A Novel In Vitro Human Macrophage Model to Study the Persistence of Mycobacterium Tuberculosis Using Vitamin D(3) and Retinoic Acid Activated THP-1 Macrophages. Front Microbiol (2011) 2:67. 10.3389/fmicb.2011.00067 21747789 PMC3128978

[88] Scordo JM Knoell DL Torrelles JB. Alveolar Epithelial Cells in Mycobacterium Tuberculosis Infection: Active Players or Innocent Bystanders? J Innate Immun (2016) 8:3–14. 10.1159/000439275 26384325 PMC4724319

[89] Vir P Gupta D Agarwal R Verma I. Immunomodulation of Alveolar Epithelial Cells by Mycobacterium Tuberculosis Phosphatidylinositol Mannosides Results in Apoptosis. APMIS (2014) 122:268–82. 10.1111/apm.12141 23919648

[90] Bernard O Jeny F Uzunhan Y Dondi E Terfous R Label R. Mesenchymal Stem Cells Reduce Hypoxia-Induced Apoptosis in Alveolar Epithelial Cells by Modulating HIF and ROS Hypoxic Signaling. Am J Physiol Lung Cell Mol Physiol (2018) 314:L360–L71. 10.1152/ajplung.00153.2017 29167125

[91] Rivas-Santiago B Torres-Juarez F. Antimicrobial Peptides for the Treatment of Pulmonary Tuberculosis, Allies or Foes? Curr Pharm Des (2018) 24:1138–47. 10.2174/1381612824666180327162357 29589538

[92] Harman RM Yang S He MK Van de Walle GR. Antimicrobial Peptides Secreted by Equine Mesenchymal Stromal Cells Inhibit the Growth of Bacteria Commonly Found in Skin Wounds. Stem Cell Res Ther (2017) 8:157. 10.1186/s13287-017-0610-6 28676123 PMC5496374

[93] Chow L Johnson V Impastato R Coy J Strumpf A Dow S. Antibacterial Activity of Human Mesenchymal Stem Cells Mediated Directly by Constitutively Secreted Factors and Indirectly by Activation of Innate Immune Effector Cells. Stem Cells Transl Med (2020) 9:235–49. 10.1002/sctm.19-0092 PMC6988770 31702119

[94] Sutton MT Fletcher D Ghosh SK Weinberg A van Heeckeren R Kaur S. Antimicrobial Properties of Mesenchymal Stem Cells: Therapeutic Potential for Cystic Fibrosis Infection, and Treatment. Stem Cells Int (2016) 2016:5303048. 10.1155/2016/5303048 26925108 PMC4746399

[95] Mizushima N. Autophagy: Process and Function. Genes Dev (2007) 21:2861–73. 10.1101/gad.1599207 18006683

[96] Mihalache CC Simon HU. Autophagy Regulation in Macrophages and Neutrophils. Exp Cell Res (2012) 318:1187–92. 10.1016/j.yexcr.2011.12.021 22245582

[97] Phadwal K Watson AS Simon AK. Tightrope Act: Autophagy in Stem Cell Renewal, Differentiation, Proliferation, and Aging. Cell Mol Life Sci (2013) 70:89–103. 10.1007/s00018-012-1032-3 22669258 PMC3535400

[98] Cen S Wang P Xie Z Yang R Li J Liu Z. Autophagy Enhances Mesenchymal Stem Cell-Mediated CD4(+) T Cell Migration and Differentiation Through CXCL8 and TGF-Beta1. Stem Cell Res Ther (2019) 10:265. 10.1186/s13287-019-1380-0 31443687 PMC6708254

[99] Datta M Via LE Chen W Baish JW Xu L Barry CE 3rd. Mathematical Model of Oxygen Transport in Tuberculosis Granulomas. Ann BioMed Eng (2016) 44:863–72. 10.1007/s10439-015-1415-3 PMC4795989 26253038

[100] Xu QQ Ge RL Sun HY Wang LS. [Research Advance on Promoting Angiogenesis of Mesenchymal Stem Cells-Review]. Zhongguo Shi Yan Xue Ye Xue Za Zhi (2017) 25:1267–70. 10.7534/j.issn.1009-2137.2017.04.056 28823307

[101] Ceccariglia S Cargnoni A Silini AR Parolini O. Autophagy: A Potential Key Contributor to the Therapeutic Action of Mesenchymal Stem Cells. Autophagy (2020) 16:28–37. 10.1080/15548627.2019.1630223 31185790 PMC6984485

[102] Hsu D Irfan M Jabeen K Iqbal N Hasan R Migliori GB. Post Tuberculosis Treatment Infectious Complications. Int J Infect Dis (2020) 92S:S41–5. 10.1016/j.ijid.2020.02.032 32114203

[103] Pasipanodya JG McNabb SJ Hilsenrath P Bae S Lykens K Vecino E. Pulmonary Impairment After Tuberculosis and Its Contribution to TB Burden. BMC Public Health (2010) 10:259. 10.1186/1471-2458-10-259 20482835 PMC2894771

[104] Ralph AP Kenangalem E Waramori G Pontororing GJ Sandjaja Tjitra E. High Morbidity During Treatment and Residual Pulmonary Disability in Pulmonary Tuberculosis: Under-Recognised Phenomena. PloS One (2013) 8:e80302. 10.1371/journal.pone.0080302 24312209 PMC3843655

[105] de la Mora IL Martinez-Oceguera D Laniado-Laborin R. Chronic Airway Obstruction After Successful Treatment of Tuberculosis and Its Impact on Quality of Life. Int J Tuberc Lung Dis (2015) 19:808–10. 10.5588/ijtld.14.0983 26056106

[106] Amaral AF Coton S Kato B Tan WC Studnicka M Janson C. Tuberculosis Associates With Both Airflow Obstruction and Low Lung Function: BOLD Results. Eur Respir J (2015) 46:1104–12. 10.1183/13993003.02325-2014 PMC4594762 26113680

[107] Aslam M Baveja R Liang OD Fernandez-Gonzalez A Lee C Mitsialis SA. Bone Marrow Stromal Cells Attenuate Lung Injury in a Murine Model of Neonatal Chronic Lung Disease. Am J Respir Crit Care Med (2009) 180:1122–30. 10.1164/rccm.200902-0242OC PMC2784417 19713447

[108] van Haaften T Byrne R Bonnet S Rochefort GY Akabutu J Bouchentouf M. Airway Delivery of Mesenchymal Stem Cells Prevents Arrested Alveolar Growth in Neonatal Lung Injury in Rats. Am J Respir Crit Care Med (2009) 180:1131–42. 10.1164/rccm.200902-0179OC PMC3269236 19713449

[109] Li J Zhou J Zhang D Song Y She J Bai C. Bone Marrow-Derived Mesenchymal Stem Cells Enhance Autophagy via PI3K/AKT Signalling to Reduce the Severity of Ischaemia/Reperfusion-Induced Lung Injury. J Cell Mol Med (2015) 19:2341–51. 10.1111/jcmm.12638 PMC4594676 26177266

[110] Gupta N Krasnodembskaya A Kapetanaki M Mouded M Tan X Serikov V. Mesenchymal Stem Cells Enhance Survival and Bacterial Clearance in Murine Escherichia Coli Pneumonia. Thorax (2012) 67:533–9. 10.1136/thoraxjnl-2011-201176 PMC3358432 22250097

[111] Gupta N Sinha R Krasnodembskaya A Xu X Nizet V Matthay MA. The TLR4-PAR1 Axis Regulates Bone Marrow Mesenchymal Stromal Cell Survival and Therapeutic Capacity in Experimental Bacterial Pneumonia. Stem Cells (2018) 36:796–806. 10.1002/stem.2796 29396891 PMC5918231

[112] Masterson C Devaney J Horie S O’Flynn L Deedigan L Elliman S. Syndecan-2-Positive, Bone Marrow-Derived Human Mesenchymal Stromal Cells Attenuate Bacterial-Induced Acute Lung Injury and Enhance Resolution of Ventilator-Induced Lung Injury in Rats. Anesthesiology (2018) 129:502–16. 10.1097/ALN.0000000000002327 29979191

[113] Zhu YG Feng XM Abbott J Fang XH Hao Q Monsel A. Human Mesenchymal Stem Cell Microvesicles for Treatment of Escherichia Coli Endotoxin-Induced Acute Lung Injury in Mice. Stem Cells (2014) 32:116–25. 10.1002/stem.1504 PMC3947321 23939814

[114] Phinney DG Di Giuseppe M Njah J Sala E Shiva S St Croix CM. Mesenchymal Stem Cells Use Extracellular Vesicles to Outsource Mitophagy and Shuttle microRNAs. Nat Commun (2015) 6:8472. 10.1038/ncomms9472 26442449 PMC4598952

[115] Cao H Wang C Chen X Hou J Xiang Z Shen Y Han X. Inhibition of Wnt/beta-catenin Signaling Suppresses Myofibroblast Differentiation of Lung Resident Mesenchymal Stem Cells and Pulmonary Fibrosis. Sci Rep (2018) 8:13644. 10.1038/s41598-018-28968-9 30206265 PMC6134002

[116] Chen S Cui G Peng C Lavin MF Sun X Zhang E. Transplantation of Adipose-Derived Mesenchymal Stem Cells Attenuates Pulmonary Fibrosis of Silicosis via Anti-Inflammatory and Anti-Apoptosis Effects in Rats. Stem Cell Res Ther (2018) 9:110. 10.1186/s13287-018-0846-9 29673394 PMC5909257

[117] Li X Wang Y An G Liang D Zhu Z Lian X. Bone Marrow Mesenchymal Stem Cells Attenuate Silica-Induced Pulmonary Fibrosis via Paracrine Mechanisms. Toxicol Lett (2017) 270:96–107. 10.1016/j.toxlet.2017.02.016 28232222

[118] Nenasheva T Nikolaev A Diykanov D Sukhanova A Tcyganov E Panteleev A. The Introduction of Mesenchymal Stromal Cells Induces Different Immunological Responses in the Lungs of Healthy and M. Tuberculosis Infected Mice. PloS One (2017) 12:e0178983. 10.1371/journal.pone.0178983 28594940 PMC5464766

[119] Erokhin VV Vasil’eva IA Konopliannikov AG Chukanov VI Tsyb AF Bagdasarian TR. [Systemic Transplantation of Autologous Mesenchymal Stem Cells of the Bone Marrow in the Treatment of Patients With Multidrug-Resistant Pulmonary Tuberculosis]. Probl Tuberk Bolezn Legk (2008) 10:3–6. 19086127

[120] Skrahin A Ahmed RK Ferrara G Rane L Poiret T Isaikina Y. Autologous Mesenchymal Stromal Cell Infusion as Adjunct Treatment in Patients With Multidrug and Extensively Drug-Resistant Tuberculosis: An Open-Label Phase 1 Safety Trial. Lancet Respir Med (2014) 2:108–22. 10.1016/S2213-2600(13)70234-0 24503266

[121] Skrahin A Jenkins HE Hurevich H Solodovnikova V Isaikina Y Klimuk D. Effectiveness of a Novel Cellular Therapy to Treat Multidrug-Resistant Tuberculosis. Int J Mycobacteriol (2016) 5(Suppl 1):S23. 10.1016/j.ijmyco.2016.11.018 28043573

[122] Yudintceva NM Bogolyubova IO Muraviov AN Sheykhov MG Vinogradova TI Sokolovich EG. Application of the Allogenic Mesenchymal Stem Cells in the Therapy of the Bladder Tuberculosis. J Tissue Eng Regener Med (2018) 12:e1580–e93. 10.1002/term.2583 28990734

[123] Poggi A Zocchi MR. Immunomodulatory Properties of Mesenchymal Stromal Cells: Still Unresolved "Yin and Yang". Curr Stem Cell Res Ther (2019) 14:344–50. 10.2174/1574888X14666181205115452 30516112

[124] Mushahary D Spittler A Kasper C Weber V Charwat V. Isolation, Cultivation, and Characterization of Human Mesenchymal Stem Cells. Cytometry A (2018) 93:19–31. 10.1002/cyto.a.23242 29072818

[125] Burja B Barlic A Erman A Mrak-Poljsak K Tomsic M Sodin-Semrl S. Human Mesenchymal Stromal Cells From Different Tissues Exhibit Unique Responses to Different Inflammatory Stimuli. Curr Res Transl Med (2020) 68:217–24. 10.1016/j.retram.2020.05.006 32843323

[126] Lukomska B Stanaszek L Zuba-Surma E Legosz P Sarzynska S Drela K. Challenges and Controversies in Human Mesenchymal Stem Cell Therapy. Stem Cells Int (2019) 2019:9628536. 10.1155/2019/9628536 31093291 PMC6481040

[127] Cadena AM Fortune SM Flynn JL. Heterogeneity in Tuberculosis. Nat Rev Immunol (2017) 17:691–702. 10.1038/nri.2017.69 28736436 PMC6247113

[128] Shahir M Mahmoud Hashemi S Asadirad A Varahram M Kazempour-Dizaji M Folkerts G. Effect of Mesenchymal Stem Cell-Derived Exosomes on the Induction of Mouse Tolerogenic Dendritic Cells. J Cell Physiol (2020) 235:7043–55. 10.1002/jcp.29601 PMC7496360 32043593

[129] Burrello J Monticone S Gai C Gomez Y Kholia S Camussi G. Stem Cell-Derived Extracellular Vesicles and Immune-Modulation. Front Cell Dev Biol (2016) 4:83. 10.3389/fcell.2016.00083 27597941 PMC4992732

[130] Stephen J Bravo EL Colligan D Fraser AR Petrik J Campbell JD. Mesenchymal Stromal Cells as Multifunctional Cellular Therapeutics - a Potential Role for Extracellular Vesicles. Transfus Apher Sci (2016) 55:62–9. 10.1016/j.transci.2016.07.011 27452645

[131] Gupta UD Katoch VM. Animal Models of Tuberculosis. Tuberculosis (Edinb) (2005) 85:277–93. 10.1016/j.tube.2005.08.008 16249122

[132] Kapoor N Pawar S Sirakova TD Deb C Warren WL Kolattukudy PE. Human Granuloma In Vitro Model, for TB Dormancy and Resuscitation. PloS One (2013) 8:e53657. 10.1371/journal.pone.0053657 23308269 PMC3538642

[133] Braian C Svensson M Brighenti S Lerm M Parasa VR. A 3d Human Lung Tissue Model for Functional Studies on Mycobacterium Tuberculosis Infection. J Vis Exp (2015) 104:53084. 10.3791/53084 PMC4692636 26485646

[134] Benmerzoug S Quesniaux VFJ. Bioengineered 3D Models for Studying Human Cell-Tuberculosis Interactions. Trends Microbiol (2017) 25:245–46. 10.1016/j.tim.2017.02.009 28284875

[135] Dobos KM Spotts EA Quinn FD King CH. Necrosis of Lung Epithelial Cells During Infection With Mycobacterium Tuberculosis Is Preceded by Cell Permeation. Infect Immun (2000) 68:6300–10. 10.1128/iai.68.11.6300-6310.2000 PMC97713 11035739

[136] Puissegur MP Botanch C Duteyrat JL Delsol G Caratero C Altare F. An In Vitro Dual Model of Mycobacterial Granulomas to Investigate the Molecular Interactions Between Mycobacteria and Human Host Cells. Cell Microbiol (2004) 6:423–33. 10.1111/j.1462-5822.2004.00371.x 15056213

[137] Auguste P Tsertsvadze A Pink J Court R McCarthy N Sutcliffe P. Comparing Interferon-Gamma Release Assays With Tuberculin Skin Test for Identifying Latent Tuberculosis Infection That Progresses to Active Tuberculosis: Systematic Review and Meta-Analysis. BMC Infect Dis (2017) 17:200. 10.1186/s12879-017-2301-4 28274215 PMC5343308

[138] Rao M Ippolito G Mfinanga S Ntoumi F Yeboah-Manu D Vilaplana C. Latent TB Infection (LTBI) - Mycobacterium Tuberculosis Pathogenesis and the Dynamics of the Granuloma Battleground. Int J Infect Dis (2019) 80S:S58–61. 10.1016/j.ijid.2019.02.035 30822547

[139] Pai M Behr M. Latent Mycobacterium Tuberculosis Infection and Interferon-Gamma Release Assays. Microbiol Spectr (2016) 4:24. 10.1128/microbiolspec.TBTB2-0023-2016 27763261

[140] Xu JC Li ZY Chen XN Shi CL Wu MY Chen H. More Significance of TB-IGRA Except for the Diagnose of Tuberculosis. J Clin Lab Anal (2018) 32:e22183. 10.1002/jcla.22183 PMC6817251 28220979

[141] Sharma SK Vashishtha R Chauhan LS Sreenivas V Seth D. Comparison of TST and IGRA in Diagnosis of Latent Tuberculosis Infection in a High TB-Burden Setting. PloS One (2017) 12:e0169539. 10.1371/journal.pone.0169539 28060926 PMC5218498

[142] Balcells ME Ruiz-Tagle C Tiznado C Garcia P Naves R. Diagnostic Performance of GM-CSF and IL-2 in Response to Long-Term Specific-Antigen Cell Stimulation in Patients With Active and Latent Tuberculosis Infection. Tuberculosis (Edinb) (2018) 112:110–19. 10.1016/j.tube.2018.08.006 30205963

[143] Lee JY Jeong I Joh JS Jung YW Sim SY Choi B. Differential Expression of CD57 in Antigen-Reactive CD4+ T Cells Between Active and Latent Tuberculosis Infection. Clin Immunol (2015) 159:37–46. 10.1016/j.clim.2015.04.011 25931385

[144] Rakshit S Adiga V Nayak S Sahoo PN Sharma PK van Meijgaarden KE. Circulating Mycobacterium Tuberculosis DosR Latency Antigen-Specific, Polyfunctional, Regulatory IL10(+) Th17 CD4 T-Cells Differentiate Latent From Active Tuberculosis. Sci Rep (2017) 7:11948. 10.1038/s41598-017-10773-5 28931830 PMC5607261

[145] Xuan WX Lu TT Wang Z An YX Zhang XJ. Diagnostic Significance of Mycobacterium Tuberculosis T-Cell Assays for Active Tuberculosis. Chin Med J (Engl) (2017) 130:811–16. 10.4103/0366-6999.202738 PMC5381315 28345545

[146] Luo Y Xue Y Guo X Lin Q Mao L Tang G. Diagnostic Accuracy of T-SPOT.TB Assay for Tuberculous Meningitis: An Updated Meta-Analysis. Front Neurol (2020) 11:866. 10.3389/fneur.2020.00866 33013621 PMC7494808

[147] Banerjee R Balaji M Sasikala M Anuradha S Rao GV Nageshwar Reddy D. Granulomas of Intestinal Tuberculosis and Crohn’s Disease Can Be Differentiated by CD73 Cell Surface Marker Expression: A Pilot Study. Dig Dis Sci (2013) 58:2301–7. 10.1007/s10620-013-2667-0 23625284

[148] Watermeyer GA Locketz M. CD73 Expression in Tissue Granulomas in Distinguishing Intestinal Tuberculosis From Crohn’s Disease in a South African Cohort. Scand J Gastroenterol (2018) 53:1217–21. 10.1080/00365521.2018.1503326 30295112

[149] Cho JH. The Genetics and Immunopathogenesis of Inflammatory Bowel Disease. Nat Rev Immunol (2008) 8:458–66. 10.1038/nri2340 18500230

